# Modulating the Release Kinetics of Natural Product Actinomycin from Bacterial Nanocellulose Films and Their Antimicrobial Activity

**DOI:** 10.3390/bioengineering11080847

**Published:** 2024-08-19

**Authors:** Katarzyna Zimowska, Vuk Filipovic, Jasmina Nikodinovic-Runic, Jelena Simic, Tatjana Ilic-Tomic, Malgorzata Zimowska, Jacek Gurgul, Marijana Ponjavic

**Affiliations:** 1 Institute of Molecular Genetics and Genetic Engineering, University of Belgrade, Vojvode Stepe 444a, 11042 Belgrade, Serbia; kzimowska8@gmail.com (K.Z.); vfilipovic@imgge.bg.ac.rs (V.F.); jasmina.nikodinovic@imgge.bg.ac.rs (J.N.-R.); jelena_lazic@imgge.bg.ac.rs (J.S.); tatjanait@imgge.bg.ac.rs (T.I.-T.); 2 Jerzy Haber Institute of Catalysis and Surface Chemistry, Polish Academy of Sciences, Niezapominajek 8, 30-239 Krakow, Poland; nczimows@cyf-kr.edu.pl (M.Z.); jacek.gurgul@ikifp.edu.pl (J.G.)

**Keywords:** actinomycin, bacterial nanocellulose, TEMPO oxidation, drug release

## Abstract

The present study aimed to create a more sustainable and controlled delivery system based on natural biopolymer bacterial nanocellulose (BNC) and bacterial natural product actinomycin (Act), with the applicative potential in the biomedical field. In order to provide improved interaction between BNC and the active compound, and thus to modulate the release kinetics, the TEMPO oxidation of BNC support was carried out. A mix of actinomycins from bacterial fermentation (ActX) were used as natural antimicrobial agents with an established bioactivity profile and clinical use. BNC and TEMPO-oxidized BNC films with incorporated active compounds were obtained and analyzed by FTIR, SEM, XPS, and XRD. The ActX release profiles were determined in phosphate-buffer solution, PBS, at 37 °C over time. FTIR analysis confirmed the improved incorporation and efficiency of ActX adsorption on oxidized BNC due to the availability of more active sites provided by oxidation. SEM analysis indicated the incorporation of ActX into the less-dense morphology of the TEMPO-oxidized BNC in comparison to pure BNC. The release kinetics of ActX were significantly affected by the BNC structure, and the activated BNC sample indicated the sustained release of active compounds over time, corresponding to the Fickian diffusion mechanism. Antimicrobial tests using *Staphylococcus aureus* NCTC 6571 confirmed the potency of this BNC-based system for biomedical applications, taking advantage of the capacity of modified BNC to control and modulate the release of bioactive compounds.

## 1. Introduction

Cellulose is the most plentiful polymeric material found in nature, and it can be obtained from the cell walls of plants and woods, bacteria, algae, and tunicates [1]. All these celluloses share the same chemical structures, consisting of *D*-glucose units connected through (1 → 4) glycoside bonds in the polymer chain, but the biosynthetic route of production favors bacterial nanocellulose (BNC) over other structural analogs. Microbial fermentation, as a renewable and highly sustainable route for BNC production, is more than desirable in producing cost-effective cellulose material [2]. It is produced by Gram-negative acetic acid bacteria, *Komagataeibacter xylinum* and *Gluconacetobacter hansenii*, as well as by bacteria from genera such as *Aerobacter*, *Enterobacter*, *Coronobacter*, *Achromobacter*, *Clostridium*, *Rhizobium*, *Pseudomonas*, and *Alcaligenes* [3]. BNC was efficiently produced from a variety of substrates, including degraded biopolymers such as PLA [4], a mixture of petrochemical and biopolymers [5], but also lignocellulosic residues obtained as by-products of the forest industry [6] and agro-industrial waste [7], making it a highly sustainable material. The remarkably high purity of the BNC in comparison to plant celluloses, with no lignin, hemicellulose, pectin, or extractives, combined with its exceptional physical and chemical properties, positions the bacterial nanocellulose as a leading material for various applications [8]. BNC offers adaptable surface chemistry, a high surface area, biocompatibility, and biodegradability [9]. BNC also has a unique morphology of nanoporous and fibrous structures, high crystallinity, thermal and mechanical stability, aspect ratio, optical transparency, and the ability to adsorb and retain liquids [10,11]. BNC is a non-toxic, non-inflammable, renewable, and biodegradable biomaterial that does not exhibit immunogenic or allergenic effects and shows good compatibility with living tissue, making BNC the material of choice for biomedical purposes such as artificial skin, antimicrobial materials for wound healing, scaffolds for tissue regeneration, artificial blood vessels, cell therapy, drug delivery systems, and others [12,13,14]. BNC is also used as membranes for water treatment [15], in the food industry [6], food packaging [16], and electrocatalysis [17]. Despite its unique material characteristics, the number of drug-loaded BNC-based carriers in clinical trials or on the market remains low; however, its appeal has grown due to its animal-free origin and versatility in custom-designed shapes like fleeces, foils, spheres, tubes, fibrous aggregates, or irregularly formed pulp [18,19].

Actinomycin (Act), a polypeptide antibiotic derived from the *Streptomyces* genus, was the first antibiotic approved by the FDA for cancer treatment back in 1964 [20]. Act structure contains a planar 2-aminophenoxazin-3-one chromophore (actinocin), along with two planar cyclic pentapeptide lactones, which can specifically intercalate to GC-rich fragments of DNA [21]. Therefore, it inhibits RNA polymerase progression and is extensively utilized as a transcription inhibitor [22,23]. It has been used in the treatment of testis, gestational trophoblastic and ovarian cancers, Ewing’s sarcoma, rhabdomyosarcoma, and Wilms’ tumor [23]. Actinomycin D (also known as C1) is usually produced as the primary component in a mixture with actinomycins C2 and C3, which vary by specific amino acid substitutions within their peptide chains [24].

To enhance the efficiency of the transport of drug molecules to target cells, drug delivery systems are used that combine the active ingredient with a carrier. These systems aim to minimize side effects, improve drug bioavailability and efficacy, and facilitate rapid healing [25,26,27]. Drug carriers play a crucial role in controlled-release applications, providing prolonged drug delivery and keeping drug concentration within therapeutic limits. Therefore, it is essential to find carrier/drug systems that are natural, non-toxic, inexpensive, and able to maintain good biological activity with minimal side effects. Nanosized controlled drug delivery systems of natural origin, such as BNC, are highly effective in targeted drug delivery, minimizing side effects, and enhancing drug bioavailability [10]. Bacterial nanocellulose is recognized as one of the best platforms when it comes to biopolymers for drug delivery applications in different forms: nanoparticles, tablets, aerogels, hydrogels, membranes, due to its high surface area, mechanical strength, aspect ratio, absence of immunogenicity, and remarkable biocompatibility [28]. The BNC merits in controlled release are in efficient transport of the incorporated drug locally, while its high surface-to-volume ratio is beneficial for good cell adhesion and absorption [29,30]. Specifically, BNC was used for the immobilization of different therapeutic agents (caffeine, ibuprofen, lidocaine, diclofenac, cisplatine [9,31]) delivered through the various routes (transdermal, local, oral). Recent studies demonstrated the antimicrobial potential of BNC by functionalization with polihexanide and povidone–iodine [32], nisin [33], and bromelain and nisin [34], confirming the capacity of BNC to be a suitable carrier for bioactive molecules. To control the drug release properties and compatibility with various bioactive compounds, a range of simple chemical modifications were performed on BNC, including oxidation, esterification, etherification, amidation, and phosphorylation [18,35]. Oxidation of the hydroxyl groups available in BNC introduced a negative charge on the material’s surface, enabling it to form electrostatic interactions with antibiotic amoxicillin, nanoparticles, and metal ions [36,37,38]. The low production cost, easy availability, biocompatibility, outstanding mechanical properties, and non-toxicity put BNC apart from other biopolymers recently used in biomedical applications. Moreover, BNC is considered a promising alternative to the commonly used carbon-based polymer matrix utilized in drug delivery systems [30].

In this context, the present study aims to develop a more efficient sustained release of antimicrobial agent actinomycin D mixture (ActX) by selecting BNC in oxidized and non-oxidized forms as a carrier of the ActX. The new activated materials obtained by the adsorption of ActX on BNC and TEMPO-mediated oxidized BNC have been demonstrated for the first time. The focus is on the physicochemical characterization of the new drug delivery system and the investigation of its release kinetics and antimicrobial activity of the new materials.

## 2. Materials and Methods

### 2.1. Materials

TEMPO, 2,2,6,6-tetramethylpiperidine 1-oxyl (98% purity), citric acid, and ethyl-acetate (EtOAc) were acquired from Sigma-Aldrich (Munich, Germany). Sodium bromide (NaBr), sodium hydroxide (NaOH), sodium hypochlorite (NaClO), sodium hydrogen phosphate heptahydrate (Na_2_HPO_4_·7H_2_O), and *n*-hexane (Hex) were obtained from ACROS Organics (Geel, Belgium). Ethanol (EtOH) was obtained from Centrohem (Stara Pazova, Serbia), and hydrochloric acid (36% concentration) was obtained from Zorka Pharma (Sabac, Serbia). Glucose, bactopeptone, and yeast extract were purchased from Biolife (Milan, Italy), and potassium hydroxide (KOH) and methanol (MeOH) were obtained from Fisher Chemicals (Fisher Scientific, Loughborough, Leicestershire, UK).

### 2.2. BNC Production

Bacterial nanocellulose (BNC) was produced by cultivating the *Komagataeibacter medellinensis* ID13488 strain (CECT 8140 from the Spanish Type Culture Collection) under static conditions in the Hestrin–Schramm (HS) medium. The cultivation was performed and purified according to the previously adopted procedure [39]. The produced BCN sheets were freeze-dried by lyophilization, resulting in highly porous white BNC films after 24 h (using a Martin Christ Alpha 1–2 LDplus, Osterode am Harz, Germany). BNC yield was determined using Equation (1):

(1) Yield mgmL=mBNCVculture medium

where *m*_BNC_ represents the dry weight of purified BNC membranes per 1 mL of HS medium.

### 2.3. TEMPO Oxidation of BNC

A freeze-dried BNC membrane (100 mg) sheet was cut into small pieces (3 cm × 1 cm) and suspended in 30 mL of deionized water, followed by the addition of 50 mg of NaBr, 4.0 mg of TEMPO, and 2.3 mL of 5% solution of NaClO (15 mmol/g cellulose) to the reaction mixture. The reaction mixture was stirred for 6 h at room temperature. The reaction was stopped by adding 5 mL of EtOH, and the obtained *ox*-BNC (oxidized BNC) was washed three times with deionized water to remove unreacted residues. The yield of the reaction was 84.3 mg (84.0%)

### 2.4. Production and Purification of Actinomycin D Mixture (ActX)

*Streptomyces* sp. BV365 strain was grown on mannitol–soy flour (MSF; 20 g/L mannitol, 20 g/L soybean flour, 20 g/L agar) plates at 30 °C for 7 days. Vegetative medium (15 g/L maltose, 8 g/L tryptic soy broth, 4 g/L yeast extract, 2 g/L CaCO_3_) in Erlenmeyer flasks (1:5, culture-to-volume ratio) was inoculated using an inoculation loop full of BV365 mycelium from a fresh MSF agar plate and incubated at 180 rpm at 30 °C for 48 h.

Fermentation was performed in a bioreactor in 3 L of the MSF liquid fermentation medium (20 g/L mannitol, 20 g/L soybean flour) at 30.0 ± 2.0 °C, pH 7.0 ± 1.0 and a maximum stirring speed of 400 rpm to ensure a minimum aeration level of 30 ± 10% during the fermentation process. The pH value was maintained at 7.0 using a 10 M solution of NaOH and 20% HCl. The fermentation culture was sampled at regular intervals for the spectrophotometric monitoring of ActX production (*A*_440_).

*Streptomyces* sp. BV365 culture was centrifuged at 6037 rcf (relative centrifugal force) at 4 °C for 20 min. ActX was extracted from the biomass with EtOAc/1% HCl three times by vortexing for 5 min. Biomass extraction yielded 775 mg/L crude extract, which was purified using gravitation column chromatography performed on 57.0 g of silica gel (SiO_2_, particle size 0.040–0.063 mm). Solvent mixtures were reported as volume/volume (*v*/*v*). Elution was performed with Hex (100 mL), Hex/EtOAc (8/2, 100 mL), Hex/EtOAc (6/4, 100 mL), Hex/EtOAc (1/1, 100 mL), Hex/EtOAc (2/8, 100 mL), EtOAc (100 mL), EtOAc/MeOH (95/5, 100 mL), EtOAc/MeOH (9/1, 100 mL). The collected fractions were evaporated to yield 160 mg/L ActX.

### 2.5. Immobilization of ActX on BNC (BNC-ActX) and Oxidized BNC (ox-BNC- ActX)

BNC and *ox*-BNC hydrogel sheets (50.0 mg of dried BNC) were immersed in a solution of Actinomycin X (5.0 mg of Actinomycin X in 20 mL of MeOH, 10 wt% of active compound per dried BNC). The Erlenmeyer vessel with the mixture was covered with aluminum foil to prevent the decomposition of ActX when exposed to light, and the mixture was stirred at 37 °C at 100 rpm (revolutions per minute) for 24 h. After the specified time, the ActX solution was allowed to evaporate, and the remaining BNC-ActX and *ox*-BNC-ActX gels were dried to a constant weight. By measuring the physical masses of BNCs and ActX in feed and after ActX adsorption, it was possible to estimate the yield of the produced BNC-ActX and *ox*-BNC-ActX by applying Equation (2):

(2) $$Yield\left(\%\right)=\frac{m_{final}}{m_{feed}}\times 100$$

where *m*_final_ refers to the mass of the dried BNCs samples containing ActX and *m*_feed_ defines the starting amount of BNCs and ActX added in the reaction mixture.

The obtained yields were 81.0% and 98.0% for BNC-ActX and *ox*-BNC-ActX, respectively.

### 2.6. Efficiency of ActX Immobilization

The immobilization efficiency (%) of the active compound, ActX, was estimated by UV–Vis spectroscopy (UV-1900i, SHIMADZU, Kyoto, Japan), where the concentration of ActX was measured before immersing the BNC in ActX solution and after stopping the immersion process (24 h). To estimate these values, a calibration curve of ActX in methanol was prepared for concentrations ranging from 10 ppm to 150 ppm, following the absorbance at the wavelength of *λ* = 440 nm (*y* = 0.0133*x* + 0.0284, *R*^2^ = 0.998). The measurements were performed in triplicate to ensure the accuracy and precision of the results.

### 2.7. Characterization of BNC Samples

#### 2.7.1. Fourier Transform Infrared (FTIR) Spectroscopy Analysis

FTIR spectroscopy was used to analyze the structure of the prepared BNC samples and to detect possible interactions between ActX and BNC after immobilization. The dried samples were recorded using an IR-affinity spectrophotometer (Thermo Fisher Scientific, NICOLET iS10, Waltham, MA, USA) in attenuated total reflection (ATR) mode. All samples were analyzed at room temperature in the wavenumber range of 4000–400 cm^−1^, a scan of 4 cm^−1^, with a fixed number of scans of 32. In addition, the degree of oxidation (*DO*) of the oxidized BNC was calculated from the FTIR spectra by applying the following Equation (3):

(3) DO=0.01+0.7×I1630–1640I1029

where *I*_1630–1640_ and *I*_1029_ correspond to the intensity of the characteristic carboxyl peak and the highest absorbance peak, respectively [40,41].

#### 2.7.2. Scanning Electron Microscopy (SEM) Analysis

SEM imaging was performed using a JEOL JSM-6390LV SEM (JEOL USA Inc. Peabody, MA, USA), operating at 15 keV. Prior to recording, the samples were sputtered onto carbon tape and coated with a conductive gold layer.

#### 2.7.3. X-ray Diffraction (XRD) Analysis

The XRD analysis was used to evaluate the crystallinity of the resulting BNC layers, as well as the state of the incorporated active compound. At ambient temperature, X-ray diffraction patterns were recorded with an X’Pert PRO diffractometer (PANalytical B.V.) operating at 40 kV and 30 mA current, using Ni-filtered Cu Kα radiation (λ = 1.54 Å). Data were collected in the range of 2*θ* = 5–30°, with a step of 0.02° and a rate of one degr#ee per minute. The crystallinity index, XC, was calculated based on Equation (4):

(4) $$XC=\frac{I_{002}-I_{bl}}{I_{002}}\times 100$$

where *I*_002_—the maximum intensity of diffraction peak from (002) plane, *I*_bl_—diffraction intensity of the background taken at an angle 2*θ* between 15° and 19°.

The crystallite sizes of the investigated samples were calculated by measuring the FWHM (full width at half maximum) of the reflections using the Scherrer equation, which was carried out by X’Pert HighScore 5.1 software using the PeakFit mode.

#### 2.7.4. X-ray Photoelectron Spectroscopy (XPS) Analysis

The XPS measurements were carried out using an SES R4000 hemispherical analyzer (Gammadata Scienta, Sweden). Non-monochromatized MgK_α_ radiation (1253.6 eV) was used to generate core excitations while the X-ray tube was operated at 12 kV and an emission current of 15 mA. The energy resolution, measured as a full width at half maximum (FWHM) for the Ag 3d_5/2_ line, was 0.9 eV (pass energy 100 eV). The spectrometer was calibrated according to ISO 15472:2001 [42], and the base pressure in the analytical chamber was 1 × 10^−10^ mbar and about 1 × 10^−8^ mbar during the experiments. The condition for the samples’ recording and their further evaluation using CasaXPS 2.3.23 software were adopted from a previous work [43]. The samples were weakly conductive, so all binding energy values are corrected for C 1s line (C-C/C-H-bonds) at 285.0 eV.

### 2.8. Actinomycin Release

The release kinetics of the active compound were monitored in phosphate-buffer solution (PBS, pH = 7.4) over time at 37 °C, 180 rpm in the dark. Both BNC and ox-BNC samples containing ActX (20.0 mg) were placed in 15 mL glass vials, and 10 mL of PBS was added. At the starting hour, samples were collected after 10 min, after which the kinetics of ActX release were tracked at specific time intervals (1 h, 1.5 h, 2 h, 2.5 h, 3 h, 4 h, 5 h, 6 h, 24 h, 28 h, 48 h, 72 h, and 144 h). For each time point, 0.5 mL samples were taken and replaced with fresh aliquots of buffer. The concentration of ActX released over time was tracked by measuring the absorbance (*A*_440_) using a UV–Vis spectrophotometer (UV-1900i, SHIMADZU, Japan). A calibration curve of the ActX in PBS was made for a concentration range of 5–200 ppm (*y* = 0.0016*x* − 0.0409, *R*^2^ = 0.99). The final results of the samples collected at each time point in duplicate were presented as average values ± SD.

### 2.9. Antibacterial Activity of ActX—BNC Films

The antibacterial activity of control and ActX-impregnated BNC was evaluated by an agar well diffusion method on the *Staphylococcus aureus* NCTC 6571 reference strain obtained from the National Collection of Type Cultures. The entire surface of the LB agar plate was inoculated by spreading a volume of microbial inoculum to a final optical density (*OD*_6000_) of 0.1. Then, holes with a diameter of 6 mm were aseptically punched and filled with sample fragments (0.25 cm^2^ in 100 µL of LB medium). In parallel, sample fragments were placed directly on agar plates. The prepared agar plates were incubated at 37 °C for 24 h. Growth inhibition was assessed by clear zones around the samples.

## 3. Results and Discussion

**Figure 1:**
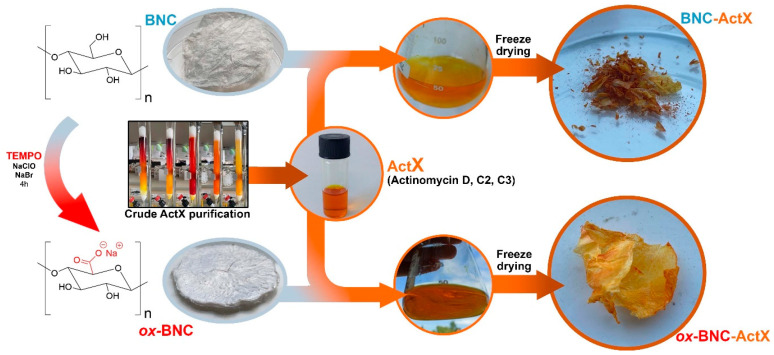
Conceptualization of active BNC samples’ (BNC-ActX and ox-BNC-ActX) preparation.

The purpose of this study was to investigate how chemical modifications affect the structure and the morphology of BNC films using a TEMPO-mediated oxidation process, but also the influence of BNC functionalization on the efficiency of immobilization/incorporation of ActX as an active compound (Figure 1). The introduction of carboxylate groups into the structure of BNC improves its functionality by increasing structural polarity, promoting better interaction with water molecules, and enhancing water retention capacity [44]. The yields of the functionalized BNCs materials were very high, 81.0% for BNC-ActX and 98.0% for *ox*-BNC-ActX, indicating better adsorption of the active compound in the case of oxidized BNC. Indeed, the estimated efficiency of adsorption for BNC-ActX was only 21.2% in comparison to the remarkably better adsorption of ActX on *ox*-BNC, 82.0%. The process of oxidation facilitated the conversion of the primary –OH functional groups on each *β*-D-glucopyranose monomer of BNC into anionic and more hydrophilic carboxylate groups (–COO^−^Na^+^), thus introducing a negative charge into the BNC structure. The introduction of –COO^−^ groups on the BNC surface directly reflected the higher susceptibility of BNC to adsorb and interact with active molecules by providing anchor points on the surface for further physical interactions and hydrogen bonding, as determined by FTIR and XPS analyses.

### 3.1. FTIR Analysis

**Figure 2:**
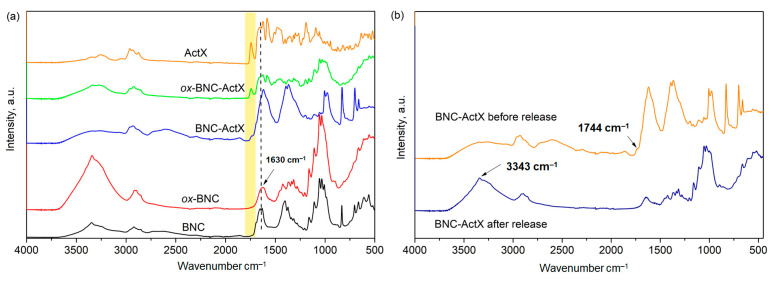
FTIR spectra of (**a**) pure BNC, oxidized BNC (*ox*-BNC), ActX containing BNC samples and ActX alone, (**b**) BNC-ActX sample before and after active compound release.

FTIR spectra were taken to confirm the structure of TEMPO-oxidized BNC and the effective binding of ActX on both BNC and *ox*-BNC (Figure 2). A clear and broad peak at 3200–3500 cm^−1^, attributed to the stretching vibrations of hydrogen-bonded hydroxyl groups, was visible in the BNC control spectrum [45], and the characteristic aliphatic C–H stretching peak (sp^3^) was observed at 2896 cm^−1^. The absorbance bands referring to the C–O and –C–O–C stretching of the *β*-glycoside bond between glucose units were detected at 970–1160 cm^−1^, but also the vibration of amorphous cellulose (stretching of the glucose ring) located at 899 cm^−1^ [46]. The band at 1630 cm^−1^ is coming from the –C=O group of residual BNC impurities such as proteins remained after the purification procedure [47] while the peak located at a wavenumber of 1653 cm^−1^ corresponds to the carboxylic groups in the ionized form –COO^−^Na^+^ after the neutralization of BNC suspension with NaOH [48]. After the oxidation by TEMPO, the characteristic absorbance peak at 1653 cm^−1^ disappeared and only a single peak at 1630 cm^−1^ was confirmed, indicating that the residues/impurities were eliminated upon oxidation, probably due to the additional washing [49]. The absence of the absorbance peak at 899 cm^−1^ indicated that the amorphous content was also reduced by oxidation with TEMPO, but the different appearance of the peak ranged from 2780 to 2970 cm^−1^ of *ox*-BNC compared to pristine BNC. The possibility of expressing the degree of oxidation (*DO*) from the intensity ratio of characteristic peaks was used to quantify the total amount of carbonyl group introduced after the TEMPO oxidation. Indeed, the *DO* value of the initial BNC was 0.099, while this value calculated for *ox*-BNC was remarkably two-fold higher at 0.21, which is consistent with the previously reported results for oxidized BNC [41]. The spectrum of pure ActX showed a characteristic absorption band from the ester carbonyl group at 1744 cm^−1^ and broad peaks at 3331 and 3260 cm^−1^ from N–H stretching vibrations [50]. Also, characteristic peaks from the amide carbonyl group at 1632 cm^−1^ (amide I band) and N–H deformation at 1560 cm^−1^ (amide II band) were visible.

The introduction of ActX into BNC and *ox*-BNC introduces changes in the bands in the FTIR spectrum, manifested as O–H and N–H ActX hydrogen bonding peaks at 3237 and 3253 cm^−1^, with a noticeably lower intensity compared to the hydrogen bonding peaks of pure BNC (Figure 2a). This may indicate the breaking of the original H-bonds of the hydroxyl group of BNC and the binding of ActX to BNC and *ox*-BNC via new H-bonds [51]. In addition, the ActX ester carbonyl peak was also present in both spectra but had a higher intensity in the *ox*-BNC-ActX spectrum compared to the BNC-ActX spectrum, where a shoulder-like band appeared (Figure 2a). This suggested the enhanced adsorption ability of *ox*-BNC due to the presence of carboxylate groups, enabling stronger interactions with ActX compared to unmodified BNC.

The difference between the BNC-ActX spectra before and after ActX release is shown in Figure 2b. The disappearance of the carbonyl group peak at 1744 cm^−1^ from the ActX and the appearance of a high-intensity peak originating from the H-bonding of O–H groups at 3343 cm^−1^, as well as the characteristic carbonyl peak at 1630 cm^−1^ inherent to BNC, have been detected. From all aforementioned results, it could be induced that all the adsorbed ActX has been completely released from BNC where it established intermolecular bonds through the hydrogen bonding, without any chemical (covalent) interactions.

### 3.2. SEM Analysis

**Figure 3:**
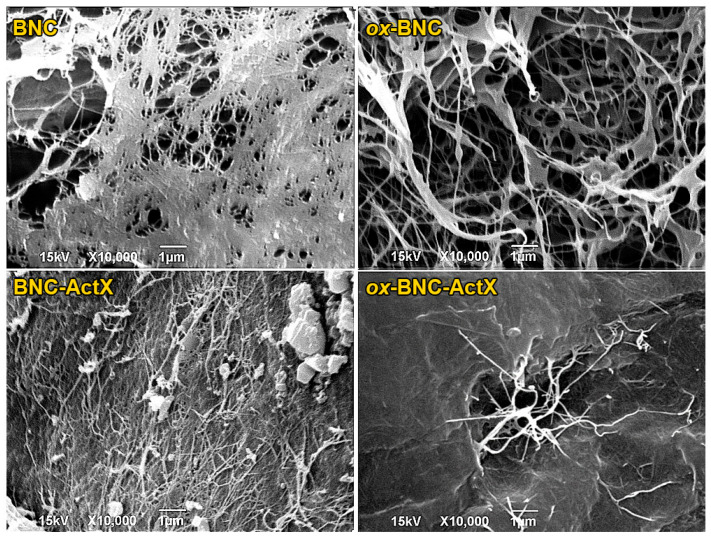
SEM micrographs of BNC, oxidized BNC (*ox*-BNC), and ActX-containing BNC samples.

The appearance and internal microstructure of the BNC and BNC-ActX film samples were observed by SEM analysis (Figure 3). The control BNC film exhibited a dense, fibrous, interconnected 3D network nanostructure. After oxidation of the BNC, the thickening of the fibers and the appearance of pores became visible on the *ox*-BNC. This change was attributed to the increased content of negatively charged carboxylate groups after TEMPO treatment, which resulted in the repulsion of BNC microfibrils. This can be beneficial for the incorporation of an active compound, especially when its adsorption occurs from an aqueous solution. Therefore, the TEMPO oxidation of BNC can both increase the surface activity and provide an effective interaction between BNC and ActX (the increase in carboxyl group content facilitates increased hydrogen bond formation between molecules) but also improve the adsorption efficiency of an active compound from solution, as shown in this study. The BNC-ActX micrograph showed that the dense morphology of the 3D BNC network was preserved, and ActX molecules were adsorbed on its surface. ActX adsorption was confirmed, but it was not perfectly uniform, as some aggregates were detected. On the other hand, a more homogenous ActX dispersion was obtained for *ox*-BNC, with the active compound molecules preferentially localized inside the BNC network rather than on the surface. The denser morphology and aggregation of *ox*-BNC fibers, induced by the combination with ActX, probably resulted from the formation of additional hydrogen bonds between carboxyl group-rich *ox*-BNC and ActX [52]. ActX molecules fill the voids of both BNC and *ox*-BNC and cover the surface of the microfibrils, which reduces the porosity of the nanocellulose networks.

### 3.3. XRD and XPS Analysis

**Figure 4:**
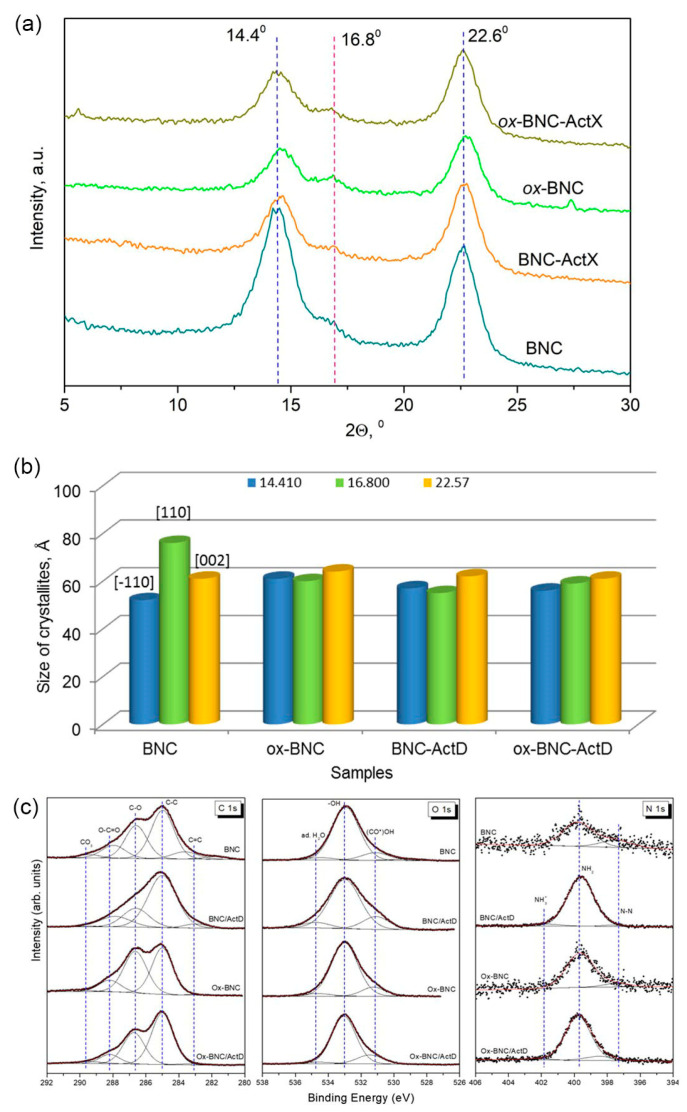
(**a**) XRD patterns of BNC and ActX containing BNC samples, (**b**) the average size of crystallites calculated for samples based on reflections at around 14.4°, 16.80°^,^ and 22.57° of 2*θ*, (**c**) high-resolution XPS spectra of C 1s, O 1s, and N 1s lines of parent and modified BNC. Dashed lines are shown as guides to the eye.

The XRD patterns of BNC, *ox*-BNC, and their ActX-activated analogs are presented in Figure 4a. In the recorded diffractograms of all samples, three characteristic diffraction peaks at 2*θ* values of 14.41°, 16.80°, and 22.57° are assigned to the crystallographic planes (–110), (110), and (002), respectively, and these peaks are dominant in well-ordered cellulose with a monoclinic crystal lattice [ICDD PDF-4+ 2015 00-056-1718], [53,54]. The 2*θ* positions of the reflections showed that the BNC products are typical of cellulose I and native cellulose. According to ICDD, diffraction peaks from (002) lattice plane at 2*θ* equal to 22.57–22.75° indicate the existence of *I*_α_ polymorph typical of bacterial and algal cellulose [53,54,55]. The intensity of the most intensive reflection attributed to the (002) plane at 2*θ* of 22.57° (*d*_hkl_ = 3.926 Å) changed, but the change was less pronounced compared to the intensity of the other two peaks, whose intensity decreased significantly after modification and activation by ActX. Oxidation of the BNC by TEMPO results in the small shift of the 14.41° and 22.57° reflections to higher 2*θ* values (14.57° and 22.75°), respectively, slightly decreasing the *d*_hkl_ values and parameters of the unit cell. Moreover, the crystallinity index of *ox*-BNC was 5% lower after the modification, probably due to the decrease in the crystalline phase and the higher content of the amorphous phase (Table 1). As a result, the average crystallite size for *ox*-BNC was slightly smaller than for native BNC, and even smaller after ActX deposition (Figure 4b). The average size of crystallites calculated for samples based on reflections at around 14.41°, 16.80°, and 22.57° of 2*θ* decreases from 6.3 to 5.8 nm, while still maintaining a high crystallinity index (Table 1).

High-resolution XPS spectra of the C 1s, O 1s, and N 1s lines were recorded to obtain information about the chemical bonds present on the surface of the BNC samples and to clarify their changes after interaction with ActX (Figure 4c). The quantitative parameters obtained from the numerical analysis of the spectra, as well as component assignments, are shown in Tables S1–S3 (Supplementary Material).

The C 1s spectra (Figure 4c) were fitted by five distinct components that were assigned as follows: (i) carbides C=C (BE < 284 eV), (ii) hydrocarbon contaminants and alkyl C–C/C–H groups (285.0 eV), (iii) C–O bonds (286.6 eV), (iv) carboxyl O–C=O and carbonyl C=O groups (288.0 eV), and (v) carbonates (BE > 289 eV). As mentioned above, the hydrocarbon contamination was used as an internal calibration for all samples (C–H peak at 285.0 eV). It is worth noting that nitrogen-containing carbon bonds can contribute to the C 1s spectrum mainly in two BE regions: at ~286.6 eV for N−C(=O)−C, N−C(=N−N) and N(=C)−N groups and at ~288.6 eV for O−C−OH and O=C−N groups [56,57,58]. TEMPO oxidation of BNC and BNC-ActX samples results in a significant reduction in the amount of carbides while increasing the component corresponding to C−O bonds. Moreover, the oxidation process causes only a slight reduction in the amount of carboxyl groups (Table S1). Samples after ActX immobilization have more alkyl groups than the parent samples.

Three main contributions distinguished in the O 1s spectra (Figure 4c) are related to (i) (CO^*^)OH and C–OH bonds (at ~531.1 eV), (ii) –OH and C=O bonds (at 533.0 eV), and (iii) aromatic carbon compounds bound to oxygen and adsorbed water (> 534.5 eV) [58]. The second component at BE of 533.0 eV is dominating for all samples (> 75%). ActX immobilization causes a slight increase in the (CO^*^)OH/C−OH component for both samples (Table S2).

The N 1s signal has been numerically deconvoluted into three components (Figure 4c). The major contribution (over 75%) can be assigned to N−C=O/N−C(O)−N and/or unprotonated amines (Table S3). Other components can be identified as coming from N−N bonds and CN ligands (at 397.7 eV) and C=N bonds and NO ligands (> 401.5 eV) [59]. Also in the latter high-BE region, a contribution from protonated amines can be expected [60]. The parent samples are characterized by a small amount of nitrogen on the surface (Figure S1), which results in poor-quality N 1s high-resolution spectra despite the prolonged measurement time. Two components can be distinguished in them, with the dominant component occurring at a binding energy of ~400 eV, which corresponds to N−C=O/N−C(O)−N bonds and unprotonated amines. It is worth mentioning here that the N−N component accounts for as much as 23% of the total spectrum of the BNC sample. Immobilization causes an increase in the amount of nitrogen on the surface and the appearance of a small N 1s component with high binding energy. This additional component attributed to C=N/NO ligands and/or protonated amines may indicate an interaction between parent and activated samples. This conclusion is also supported by the noticeable shift of the low-energy component in *ox*-BNC-ActX towards higher energies (~1 eV).

### 3.4. ActX ActX Release in PBS and Antimicrobial Activity

**Figure 5:**
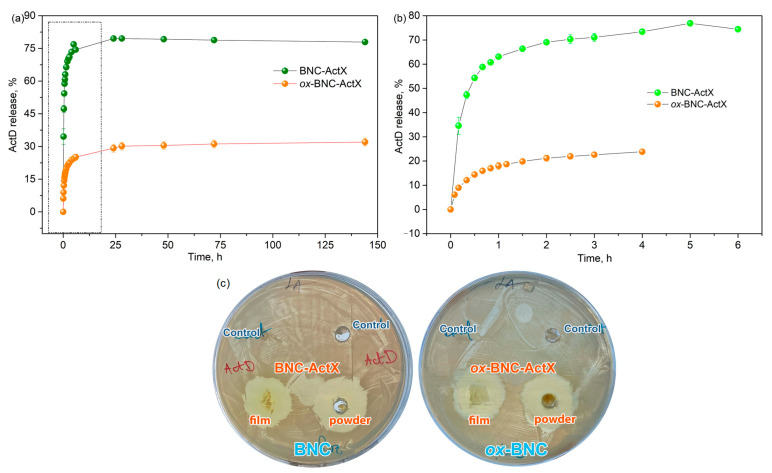
(**a**) ActX release from BNC and *ox*-BNC over time, (**b**) singled out release profiles of ActX release from BNC and *ox*-BNC in the first 6 h, (**c**) antimicrobial activity evaluated by modified agar diffusion method of BNC and *ox*-BNC hydrogels (control) and ActX impregnated hydrogels (in the form of film and pulverized) for *S. aureus* NCTC 6571.

The active compound release kinetics were monitored in PBS (pH = 7.4) over time at 37 °C, 180 rpm in the dark. Drug release profiles were investigated during six days and showed that the release of ActX from BNC reached 79% after 24 h, whereas *ox*-BNC achieved 29% after the same time (Figure 5a). In the case of *ox*-BNC, differences in the structure of BNC were observed, reflected in the release kinetics of the active compound ActX, as well as slower and more sustainable release (Figure 5b). After the initial fast release in the starting 24 h, the amount of the release ActX reached a plateau, and the concentration of the active compound stayed unchanged over time. The sustained release in topical formulations is notable as it allows for gradual bacterial inhibition, potentially improving treatment efficacy and wound healing [61]. The type of BNC—such as native wet, semi-dried, or freeze-dried—can affect the drug loading process. Compared to native wet BNC, freeze-dried BNC allows for soaking with solutions of bioactive compounds, which offers the advantages of requiring a lesser amount of the compounds, a predetermined loading dose, and increased loading efficacy [18]. Studies on the release of active compounds from BNC typically show a burst release (<15 min) [9,61,62]. This brief release time is primarily driven by diffusion through the porous, three-dimensional BNC network, facilitated by BNC’s high swelling capacity [9].

The active compound release kinetics can be analyzed by defining the characteristic kinetic parameters, such as sampling time, *τ*_x%_, referring to the time at which the specific percentage of active compound was released [63]. From the results summarized in Table 2, 50% of ActX from BNC was released after only 15 min, while the same amount of ActX was released after 55 min from *ox*-BNC, indicating a more sustainable release pattern caused by better adsorption and stronger interaction between support and ActX. This phenomenon was more expressed for the *τ*_70%_ values, and this specific amount of ActX was released from BNC after 40 min in comparison to the 180 min estimated for *ox*-BNC.

When it comes to the mathematical models that predict the mechanisms of the released active component, the release data can be fitted by various kinetic models, such as Peppas (“power-law”) [64] and Higuchi [65]. The mathematical calculations were performed in SigmaPlot 11.0 software, and the summarized data are shown in Table 2. The best fits were obtained by applying the *Peppas* kinetic model with the high correlation coefficient calculated for both BNC-ActX and *ox*-BNC-ActX. The obtained *n* values (indicating the release mechanism), below 0.50, which is the limit for the film form (the diffusion exponent indicates the release mechanism which further depends on the type of transport and geometry), suggest that the main mechanism of ActX release was governed by *Fickian* diffusion. The constant rate of BNC-ActX was higher in comparison to *ox*-BNC-ActX, confirming the initial faster release detected for non-functionalized BNC. By analyzing the fitting data obtained with the *Higuchi* model, it can be concluded that the correlation coefficient was lower (0.8466 in comparison to 0.9618), leading to a conclusion that Peppas’ model, describing the release from nano-polymeric matrices, was more appropriate for the investigated systems. Finally, all these results indicated that the release profiles of the ActX incorporated into the BNC-based matrix can be easily modulated by the functionalization of BNC and the introduction of –COO^−^ groups, which further dictated the adsorption efficiency.

Actinomycin exhibits antibacterial activity primarily against a variety of Gram-positive bacteria, including *S. aureus*, *S. epidermidis*, and *Enterococcus faecalis*, including some of their drug-resistant strains [66]. The antibacterial properties of the tested materials (BNC and *ox*-BNC) with ActX extract were evaluated through the disk diffusion assay (Figure 5c). Growth inhibition zones around the ActX extract were detected for *S. aureus* NCTC 6571 and for both BNC samples loaded with the extract. For this purpose, the ActX-activated BNC samples in the form of film and in the shredded form (to obtain the powder) were tested, and the same antimicrobial activity was obtained. BNC and *ox*-BNC controls without the active compound did not display any antimicrobial effects. The ActX-activated silk–fibroin–polyurethane membrane against *S. aureus* has been reported, where it was shown that the antibacterial activity is not only affected by the concentration of ActX but is also highly dependent on the direct contact between bacteria and the blend membrane [67]. Additionally, ActX was used for the dyeing and finishing of silk fabric, and the antibacterial properties of dyed silk have also been confirmed at over 90%, even after 20 wash cycles [50].

## 4. Conclusions

The present study addressed the potential biomedical application of BNC activated by the bacterial natural product ActX. Chemical modification of the BNC (TEMPO) significantly improved the adsorption ability of an active compound ActX and further allowed for more controlled ActX release, beneficial in drug delivery systems. TEMPO modification of BNC affected its chemical, morphological, and crystal properties, which proved to be a useful tool for adjusting the BNC properties and release kinetics of the active compounds. FTIR analysis identified the intermolecular interactions between the active compound and matrix (hydrogen bonding), and after release, the supporting material (BNC and ox-BNC) was confirmed, indicating physical interactions between BNC and ActX. Finally, both BNC and *ox*-BNC activated by ActX showed desirable antimicrobial activities, but more sustainable and controlled release observed from the *ox*-BNC matrix favors the modified BNC over the native analog.

## Figures and Tables

**Table 1:** X-ray diffraction pattern analysis of the BNC samples.

*Sample*	2*ϴ*°			*FWHM*			*X_C002,_ %*
	(–110)	(110)	(002)	(−110)	(110)	(002)	
BNC	14.41	16.80	22.57	1.60	1.11	1.39	70.48
*ox*-BNC	14.57	16.84	22.75	1.37	1.40	1.32	65.79
BNC-ActX	14.47	16.88	22.67	1.46	1.51	1.37	69.40
*ox*-BNC-ActX	14.41	16.76	22.62	1.50	1.42	1.38	66.17

*XC*_002_%—crystallinity index.

**Table 2:** Drug release kinetics: sampling time (*τ*_50%_, *τ*_60%,_ *τ*_70%_) and released amount of drug after 24 h, *c*_24_.

Sample	*τ*_50%,_ min		*τ*_60%_, min	*τ*_70%_, min		*c*_24h_, %
BNC-ActX	15		20	40		79.5
*ox*-BNC-ActX	55		80	180		29.2
**Sample**	Peppas model			Higuchi model		Mechanism
	*k*	*n*	*R*	*k*	*R*	
BNC-ActX	16.897	0.2711	0.9870	14.049	0.8466	*Fickian* diffusion
*ox*-BNC-ActX	17.495	0.3457	0.9944	17.395	0.9618	

## Data Availability

The data presented in this study are available on request from the corresponding author.

## Supplementary Materials

The following supporting information can be downloaded at: https://www.mdpi.com/article/10.3390/bioengineering11080847/s1, Table S1. C 1s peak assignment obtained from the deconvolution of high resolution XPS spectra. Binding energies (eV) and relative area percentage (in parentheses) are listed; Table S2. O 1s peak assignment obtained from the deconvolution of high resolution XPS spectra. Binding energies (eV) and relative area percentage (in parentheses) are listed; and Table S3. N 1s peak assignment obtained from the deconvolution of high resolution XPS spectra. Binding energies (eV) and relative area percentage (in parentheses) are listed.
