# Supplementary material for: Modulating the Release Kinetics of Natural Product Actinomycin from Bacterial Nanocellulose Films and Their Antimicrobial Activity
Source: Bioengineering (Basel). 2024 Aug 19;11(8):847. doi: 10.3390/bioengineering11080847 (PMC11352114; doi:10.3390/bioengineering11080847)
Supplement: Supplementary file 1 [file bioengineering-11-00847-s001.zip › bioengineering-3147852-supplementary.pdf]

Supplementary Materials

Modulating the Release Kinetics of Natural Product Actinomycin from Bacterial Nanocellulose Films and Their Antimicrobial Activity

Katarzyna Zimowska [1], Vuk Filipovic [1], Jasmina Nikodinovic-Runic [1], Jelena Simic [1], Tatjana Ilic-Tomic [1], Malgorzata Zimowska [2], Jacek Gurgul [2] and Marijana Ponjavic [1,*]

[1] Institute of Molecular Genetics and Genetic Engineering, University of Belgrade, Serbia, Vojvode Stepe 444a, 11042 Belgrade, Serbia; kzimowska8@gmail.com (K.Z.); vfilipovic@imgge.bg.ac.rs (V.F.); jasmina.nikodinovic@imgge.bg.ac.rs (J.N.-R.); jelena_lazic@imgge.bg.ac.rs (J.S.); tatjanait@imgge.bg.ac.rs (T.I.-T.)

[2] Jerzy Haber Institute of Catalysis and Surface Chemistry, Polish Academy of Sciences, Niezapominajek 8, 30-239 Krakow, Poland; nczimows@cyf-kr.edu.pl (M.Z.); jacek.gurgul@ikifp.edu.pl (J.G.)

* Correspondence: mponjavic@imgge.bg.ac.rs

Table S1. C 1s peak assignment obtained from the deconvolution of high resolution XPS spectra. Binding energies (eV) and relative area percentage (in parentheses) are listed.

Sample	C=C	C–C/C–H	C–O	C=O/O–C=O	CO_3
BNC*	283.9<br>(7.4)	285.0<br>(45.1)	286.6<br>(29.4)	288.0<br>(12.7)	289.4<br>(2.9)
BNC-ActX	283.0<br>(3.9)	285.0<br>(61.4)	286.6<br>(20.6)	287.9<br>(13.5)	289.5<br>(0.6)
ox-BNC	283.2<br>(0.3)	285.0<br>(43.7)	286.6<br>(42.6)	288.2<br>(11.4)	290.2<br>(2.0)
ox-BNC-ActX	283.2<br>(1.5)	285.0<br>(54.5)	286.7<br>(32.7)	288.2<br>(8.9)	289.4<br>(2.4)

* additional component at 281.8 eV (2.5%) most likely comes from surface charging

Table S2. O 1s peak assignment obtained from the deconvolution of high resolution XPS spectra. Binding energies (eV) and relative area percentage (in parentheses) are listed.

Sample	(CO*)OH/C–OH	–OH/C=O	ad. H_2O O-aromatic C
BNC#	531.1<br>(12.0)	532.9<br>(81.7)	534.5<br>(3.9)
BNC-ActX	531.1<br>(17.0)	533.0<br>(75.4)	534.7<br>(7.6)
ox-BNC	531.2<br>(12.4)	533.0<br>(83.4)	534.7<br>(4.2)
ox-BNC-ActX	531.4<br>(16.2)	533.0<br>(80.5)	534.6<br>(3.3)

additional component at 529.2 eV (2.4%) most likely comes from surface charging

Table S3. N 1s peak assignment obtained from the deconvolution of high resolution XPS spectra. Binding energies (eV) and relative area percentage (in parentheses) are listed.

Sample	N−N/CN ligands	N−C=O/N-C(O)-N unprotonated amines	C=N/NO ligands protonated amines
BNC	397.5<br>(23.3)	399.8<br>(76.7)	---
BNC-ActX	397.7<br>(2.1)	399.6<br>(94.4)	401.5<br>(3.5)
ox-BNC	397.4<br>(9.1)	399.6<br>(90.9)	---
ox-BNC-ActX	398.5<br>(9.0)	399.8<br>(88.5)	402.3<br>(2.5)
